# Connectivity impairment of cerebellar and sensorimotor connector hubs in Parkinson’s disease

**DOI:** 10.1093/braincomms/fcac214

**Published:** 2022-08-20

**Authors:** Epifanio Bagarinao, Kazuya Kawabata, Hirohisa Watanabe, Kazuhiro Hara, Reiko Ohdake, Aya Ogura, Michihito Masuda, Toshiyasu Kato, Satoshi Maesawa, Masahisa Katsuno, Gen Sobue

**Affiliations:** Department of Integrated Health Sciences, Nagoya University Graduate School of Medicine, Nagoya, Aichi, 461–8673 Japan; Brain & Mind Research Center, Nagoya University, Nagoya, Aichi, 466–8550 Japan; Brain & Mind Research Center, Nagoya University, Nagoya, Aichi, 466–8550 Japan; Department of Neurology, Nagoya University Graduate School of Medicine, Nagoya, Aichi, 466-8550 Japan; Brain & Mind Research Center, Nagoya University, Nagoya, Aichi, 466–8550 Japan; Department of Neurology, Nagoya University Graduate School of Medicine, Nagoya, Aichi, 466-8550 Japan; Department of Neurology, Fujita Health University School of Medicine, Toyoake, Aichi, 470-1192 Japan; Department of Neurology, Nagoya University Graduate School of Medicine, Nagoya, Aichi, 466-8550 Japan; Department of Neurology, Fujita Health University School of Medicine, Toyoake, Aichi, 470-1192 Japan; Brain & Mind Research Center, Nagoya University, Nagoya, Aichi, 466–8550 Japan; Department of Neurology, Nagoya University Graduate School of Medicine, Nagoya, Aichi, 466-8550 Japan; Department of Neurology, Nagoya University Graduate School of Medicine, Nagoya, Aichi, 466-8550 Japan; Department of Neurology, Nagoya University Graduate School of Medicine, Nagoya, Aichi, 466-8550 Japan; Brain & Mind Research Center, Nagoya University, Nagoya, Aichi, 466–8550 Japan; Department of Neurosurgery, Nagoya University Graduate School of Medicine, Nagoya, Aichi, 466-8550 Japan; Department of Neurology, Nagoya University Graduate School of Medicine, Nagoya, Aichi, 466-8550 Japan; Brain & Mind Research Center, Nagoya University, Nagoya, Aichi, 466–8550 Japan; Aichi Medical University, Nagakute, Aichi, 480-1195 Japan

**Keywords:** connector hubs, Parkinson’s disease, resting-state networks, cerebellum, sensorimotor

## Abstract

Cognitive and movement processes involved integration of several large-scale brain networks. Central to these integrative processes are connector hubs, brain regions characterized by strong connections with multiple networks. Growing evidence suggests that many neurodegenerative and psychiatric disorders are associated with connector hub dysfunctions. Using a network metric called functional connectivity overlap ratio, we investigated connector hub alterations in Parkinson’s disease. Resting-state functional MRI data from 99 patients (male/female = 44/55) and 99 age- and sex-matched healthy controls (male/female = 39/60) participating in our cross-sectional study were used in the analysis. We have identified two sets of connector hubs, mainly located in the sensorimotor cortex and cerebellum, with significant connectivity alterations with multiple resting-state networks. Sensorimotor connector hubs have impaired connections primarily with primary processing (sensorimotor, visual), visuospatial, and basal ganglia networks, whereas cerebellar connector hubs have impaired connections with basal ganglia and executive control networks. These connectivity alterations correlated with patients’ motor symptoms. Specifically, values of the functional connectivity overlap ratio of the cerebellar connector hubs were associated with tremor score, whereas that of the sensorimotor connector hubs with postural instability and gait disturbance score, suggesting potential association of each set of connector hubs with the disorder’s two predominant forms, the akinesia/rigidity and resting tremor subtypes. In addition, values of the functional connectivity overlap ratio of the sensorimotor connector hubs were highly predictive in classifying patients from controls with an accuracy of 75.76%. These findings suggest that, together with the basal ganglia, cerebellar and sensorimotor connector hubs are significantly involved in Parkinson’s disease with their connectivity dysfunction potentially driving the clinical manifestations typically observed in this disorder.

## Teaser

Bagarinao *et al*. have identified two sets of connector hubs, brain regions critical for the flow of information across neural systems, located in the sensorimotor cortex and cerebellum exhibiting significant connectivity alterations with multiple resting-state networks in patients with Parkinson’s disease. These connectivity alterations correlated with patients’ motor symptoms.

## Graphical Abstract

**Graphical abstract:**
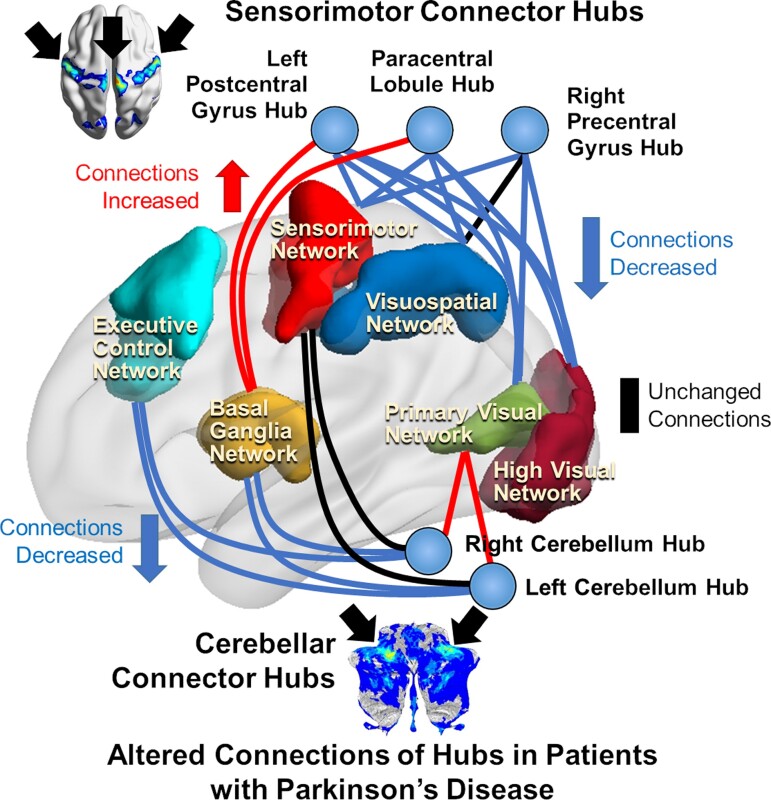

## Introduction

Neuroimaging studies have been instrumental in elucidating the functional network architecture of the human brain. Using resting-state functional magnetic resonance imaging (rsfMRI), several large-scale functional brain networks have been identified. Some of these so-called resting-state networks (RSNs) are associated with higher cognitive functions, such as the default mode,^1,2^ executive control^3^ and salience^4^ networks, as well as with primary processing, such as the sensorimotor,^5^ visual,^6^ and auditory (Aud)^7^ networks. The importance of RSNs for normal brain functions has been clearly demonstrated in several studies showing that their disruptions can be associated with neurodegenerative or psychiatric disorders.^8–14^

More recently, studies have shown that connectivity disruptions of a small number of regions in the brain, called hubs, are more critical than disruptions of non-hub regions.^15,16^ In network theory, hubs are characterized by numerous strong interconnections with other nodes in the same RSN or with other RSNs. Given its strategic connectivity, hubs are important for the coordination of information flow^17^ across neural systems. In a large meta-analysis including 26 different brain disorders,^18^ hub regions have been found to be more anatomically abnormal and generally involved in many of the disorders compared with non-hubs. Connector hubs, characterized by strong connections with multiple RSNs, are particularly important for the integration of functionally specialized systems.^19^ Disruption of connector hubs by lesion^16^ or by non-invasive transcranial magnetic stimulation^15^ had been shown to impact brain network functions and associated with widespread degradation of cognitive function.^20^

Parkinson’s disease is characterized by synaptic damage.^21–23^ Thus, analyzing pathologies at the network level is necessary to understand how impairment in one system could affect the others. As some of the disease’s clinical manifestations are increasingly being understood as complex network disorders rather than local disturbances,^24,25^ identifying affected integrative hub regions, critical for the efficient exchange of information and integration of function across large networks, is important to better understand the disease’s pathophysiology. To fully understand the mechanisms driving varying symptoms in Parkinson’s disease, it is important to examine how different neural systems, from primary processing to higher order cognitive systems, and their interactions are affected by the disease. In terms of interaction, several studies have already shown significant connectivity dysfunctions (both increases and decreases) in various brain regions including the cerebellum, sensorimotor cortex, basal ganglia, and others, in Parkinson’s disease.^26–31^ However, whether specific integrative hub regions are also involved in Parkinson’s disease has not been clearly demonstrated.

Previous studies have examined hub alterations in Parkinson’s disease using graph theory.^32–34^ In this approach, the whole brain is typically subdivided into several clusters using existing brain parcellations and used these clusters as network nodes and their connectivity with other clusters as network edges. Relevant network metrics associated with the nodal hub properties, such as nodal degree or participation coefficient,^35,36^ are then extracted and differences of these properties between patients and controls are examined. However, the identified hub regions using this approach are very limited in spatial resolution depending on the used parcellation, which typically subdivides the whole brain into just a few hundred clusters. Critical subcortical and cerebellar regions are also being excluded as most parcellations are mainly based on the cerebral cortex. In addition, metrics used to identify network hubs only measure whole-brain connections rather than connections to specific RSNs. Consequently, alterations in those metrics cannot be readily associated with any specific RSN.

The objective of this study is to identify connector hub alterations in Parkinson’s disease and their associations with the disease’s motor symptoms. We used a network metric, called functional connectivity overlap ratio (FCOR),^37^ we recently developed to examine changes in whole-brain functional connectivity (FC) in patients with Parkinson’s disease. Unlike existing approaches, FCOR can be used to identify regions with high between-network connectivity at the voxel level, enabling the identification of connector hub alterations with voxel-level resolution across the whole brain, including the cerebellum and other subcortical regions. Moreover, FCOR individually quantifies a voxel’s connection with different RSNs, thus alterations in FCOR values can be readily associated with a given RSN. These features allowed us to localize altered connector hubs to specific brain regions and explicitly identify the affected RSNs. Using rsfMRI data from patients with Parkinson’s disease and healthy controls, whole-brain FCOR maps for several well-known RSNs were generated and used to extensively examine connectivity changes across the whole brain in the patient group. Affected connector hubs were identified by examining voxels with connectivity alterations to multiple RSNs and validated using seed-based connectivity analyses.

## Materials and methods

### Participants

MRI data from 99 patients with Parkinson’s disease and 99 age- and sex-matched healthy controls were used in this study. Patients were recruited from the Department of Neurology, Nagoya University, Japan, from July 2013 to February 2019, whereas healthy controls were recruited from our ongoing Brain & Mind Research Center Aging Cohort Study,^38,39^ which started in July 2014. Details of the inclusion and exclusion criteria and matching methods between patients and controls are given in our previous paper.^40^ Motor and non-motor symptoms were evaluated using the Japanese version of the Movement Disorder Society Unified Parkinson’s Disease Rating Scale (MDS-UPDRS)^41,42^ by Japanese board-certified neurologists (K.K., H.W., and K.H.). General cognitive performance was evaluated using the Japanese version of the Addenbrooke’s Cognitive Examination-Revised^43,44^ test. Participants’ characteristics are summarized in Table 1. All neurological evaluations and MRI scans were performed during ON medication state. This study was approved by the Ethics Review Committee of Nagoya University Graduate School of Medicine and conformed to the Ethical Guidelines for Medical and Health Research Involving Human Subjects endorsed by the Japanese government. Written informed consent was obtained from all participants before joining the study.

**Table 1:** Participants’ characteristics

	Patients (N = 99)mean (SD)	Healthy controls (N = 99)mean (SD)	Two-sample *t*-testt-value (*P*-value)
Age (years)	67.39 (9.08)	67.75 (8.51)	–0.28 (0.78)
Sex (male/female)	44/55	39/60	–
Education (years)	13.58 (2.93)	13.49 (2.43)	0.21 (0.83)
ACE-R total score	91.66 (6.05)	95.22 (3.12)	–5.21 (4.80×10^–7^)
Mean frame-wise displacement	0.21 (0.10)	0.21 (0.08)	0.49 (0.63)
Duration (years)	5.2 (3.5)	–	
MDS-UPDRS			
Part I	7.39 (5.20)	–	
Part II	9.17 (6.35)	–	
Part III	28.37 (13.24)	–	
Part IV	2.25 (3.67)	–	
Hoen-Yahr	2.1 (0.67)	–	
LEDD	427.71 (299.64)	–	

ACE-R = Addenbrooke’s Cognitive Examination—Revised; LEDD = levodopa equivalent daily dosage; MDS-UPDRS = Movement Disorder Society Unified Parkinson’s Disease Rating Scale; SD = standard deviation.

### MRI data acquisition

All participants underwent MRI scanning at the Brain & Mind Research Center, Nagoya University using a Siemens Magnetom Verio (Siemens, Erlangen, Germany) 3T MRI scanner. For each participant, a high-resolution T_1_-weighted image was acquired using a 3D Magnetization-Prepared Rapid Acquisition Gradient-Echo pulse sequence^45^ with the following imaging parameters: repetition time (TR) = 2.5 s, echo time (TE) = 2.48 ms, inversion time (TI) = 900 ms, field of view (FOV) = 256 mm, 256×256 matrix dimension, in-plane voxel resolution of 1.0×1.0 mm^2^, 192 sagittal slices with a distance factor of 50% and 1 mm thickness. Aside from the T_1_-weighted image, rsfMRI data were also acquired using an ascending gradient-echo echo-planar imaging sequence with the following parameters: TR = 2.5 s, TE = 30 ms, FOV = 192 mm, 64×64 matrix dimension, flip angle = 80 degrees, 39 transverse slices with a 0.5 mm interslice interval and 3 mm thickness, and a total of 198 volumes. Participants were instructed to close their eyes, but stay awake, during the entire rsfMRI scan.

### Image preprocessing

All acquired images were preprocessed using Statistical Parametric Mapping (SPM12, Wellcome Trust Center for Neuroimaging, London, United Kingdom) software running on Matlab (R2020b, Mathworks Inc, Natick, MA, USA). The T_1_-weighted images were first segmented into component images including grey matter, white matter (WM), and CSF, among others, using the unified segmentation approach^46^ available in SPM12. During segmentation, we also generated bias-corrected T_1_-weighted image, as well as the transformation information necessary to normalize images from subject space to the Montreal Neurological Institute (MNI) space. For the rsfMRI data, the first five volumes were removed to account for the initial signal instability. The remaining images were then slice-time corrected relative to the middle slice (Slice 20), realigned relative to the mean functional image computed by first realigning the images to the first image in the series, co-registered to the bias-corrected T_1_-weighted image, and normalized to MNI space using the transformation information obtained during segmentation. The normalized images were then resampled to have a 3×3×3 mm^3^ voxel size and smoothed using a 6-mm full-width-at-half-maximum Gaussian filter. After smoothing, additional preprocessing steps were performed. Specifically, effects of head motion were regressed out using 24 motion-related regressors that included the six estimated motion parameters corresponding to translation along x-, y-, and z-axis and rotations about x-, y-, and z-axis, their derivatives, and the corresponding squares of the original estimates and their derivatives. In addition, signals from WM and CSF, estimated using the mean time course of spherical (radius = 4 mm) regions-of-interest (ROI) centred at the MNI coordinates (24, –12, 34) for WM and (20, –32, 18) for CSF, the global signal, and their derivatives were also regressed. A bandpass filter within 0.01 and 0.1 Hz were then applied to the preprocessed images. These additional preprocessing steps were implemented using in-house scripts using built-in Matlab functions.

### Functional connectivity overlap ratio

Using the preprocessed rsfMRI data, we quantified the connectivity of each voxel within the brain to different RSNs using the FCOR metric.^37,47^ For each voxel, a whole-brain seed-based connectivity analysis was performed. The resulting correlation values were then thresholded using a false discovery rate (FDR) of *q* < 0.01 to construct the voxel’s FC map. In this analysis, we only included significant positive correlations and excluded negative correlations to avoid issues with the inclusion of global signal regression during preprocessing.^48^ The FCOR value for the voxel was then estimated as the number of overlapping voxels between the constructed FC map and a reference RSN template divided by the total number of voxels within the template. Thus, the FCOR value effectively quantifies the number of connections of the voxel to the reference RSN and can range from 0, for no connection, and 1, when the entire RSN template is fully connected to the voxel. By repeating the whole process for all voxels, a whole-brain FCOR map relative to a given RSN can be constructed. FCOR values across the whole brain were then converted into *z*-scores to enable statistical comparison across participants.^49^ In this study, we evaluated FCOR maps for the 14 RSNs in the Shirer’s RSN templates,^50^ which included the dorsal and ventral default mode networks (d/v DMN), anterior and posterior salience networks (a/p Sal), left and right executive control networks (L/R ECN), visuospatial network (Visu), basal ganglia network (BG), language network (Lang), precuneus network (Prec), sensorimotor network (SMN), primary and high visual networks (p/h Vis), and Aud. For each RSN template, FCOR maps were constructed for all participant.

### Statistical analysis

#### Identification of altered connector hubs in Parkinson’s disease

To identify voxels with significant connectivity alterations to different RSNs in patients compared to controls, we used the generated FCOR maps from all participants. For each RSN, FCOR maps were compared between patients and controls using a one-sided two-sample *t*-test. We included age, sex, mean frame-wise displacement representing head motion during rsfMRI scans,^51^ and levodopa equivalent daily dose (LEDD) values as co-variates of no interest. The resulting contrast images were thresholded using *P* < 0.05, corrected for multiple comparisons using family-wise error correction at the cluster level (FWEc) with a cluster defining threshold (CDT) set to *P* = 0.001.

Candidate affected connector hubs were identified by examining voxels with significant connectivity alterations to multiple RSNs. For this, contrast maps were binarized by setting the value of voxels with significant FCOR alterations to 1 and others to 0. The binarized contrast maps from the 14 RSNs were then combined. Two conjunction maps were generated, one for each contrast (control > patients and control < patients). In these maps, the value of each voxel represents the number of RSNs where significant difference in FCOR values between control and patient groups was observed. Clusters containing peak values of the conjunction maps were then identified.

#### Verification of the identified connector hubs in healthy controls

To verify that the identified clusters were indeed connector hubs, we further performed seed-based connectivity analyses using data from healthy controls. The MNI coordinates of the clusters’ peak location were extracted and spherical ROIs centred at these coordinates with 3 mm radius were constructed. These ROIs were then used as seed regions for the succeeding seed-based connectivity analysis. For each ROI, a group FC map was constructed using a one-sided one-sample *t*-test of the generated FC maps from healthy controls and thresholded using FWEc *P* < 0.05 with a CDT of *P* = 0.001. FCOR values were then estimated using the group-level connectivity map to quantify the ROI’s connections to the different RSNs.

#### Differences in FCOR values at the identified connector hubs

Additional analyses were also performed at the connector hub level to validate the significance of the identified connector hubs. For this, mean FCOR values within the identified connector hubs were extracted from all FCOR maps and all participants. For each participant, each identified connector hub was assigned 14 mean FCOR values representing its connections to the 14 RSNs. Differences in FCOR values between patients and controls for each RSN were evaluated using a non-parametric rank sum test. Significance was assessed using FDR *q* < 0.05.

#### Classification using connector hubs’ FCOR values

We further examined the predictive power of the connector hubs’ FCOR values in classifying patients from controls. Using the extracted FCOR values from the identified connector hubs as features, linear support vector machines (SVMs) were trained for the classification. To assess the classification performance of SVMs, we used 10-fold cross validation accuracy. Connector hubs with FCOR values that lead to higher classification accuracy would indicate higher predictive values for the patient group. For this analysis, we used libSVM^52^ running on Matlab. The parameter for the linear SVM was set to the default value of 1.

#### Association between connector hubs’ FCOR values and clinical scores

Finally, we performed principal component analysis to identify the RSN connections of the identified connector hubs that contributed to the highest variance of FCOR values in the patient group. This would enable us to identify the connector hubs’ connections with the most spread in FCOR values. Using FCOR values from the identified connector hubs from the patient group, we extracted the first principal component and its corresponding component scores. The association between the extracted component scores and motor impairment scores were quantified using Pearson’s correlation coefficient. In addition, we also examined the association between individual connector hubs’ FCOR values and motor function scores. Motor function disturbances were quantified using the MDS-UPDRS Part III total score, a tremor dominant score calculated as the sum of MDS-UPDRS sub-items 2.10, 3.15, 3.16, 3.17, and 3.18, and a postural instability/gait difficulty (PIGD) score calculated as the sum of MDS-UPDRS sub-items 2.12, 2.13, 3.10, 3.11, and 3.12.^53^ In the analyses involving tremor and PIGD scores, 19 patients were excluded due to incomplete motor scores.

### Data availability

The data used in this study are not publicly available due to privacy and ethical restrictions. The data can be provided to interested researchers upon reasonable request to the corresponding author and subject for approval from the Ethics Review Committee of Nagoya University Graduate School of Medicine.

## Results

### Pattern of connectivity alterations in patients with Parkinson’s disease

**Figure 1:**
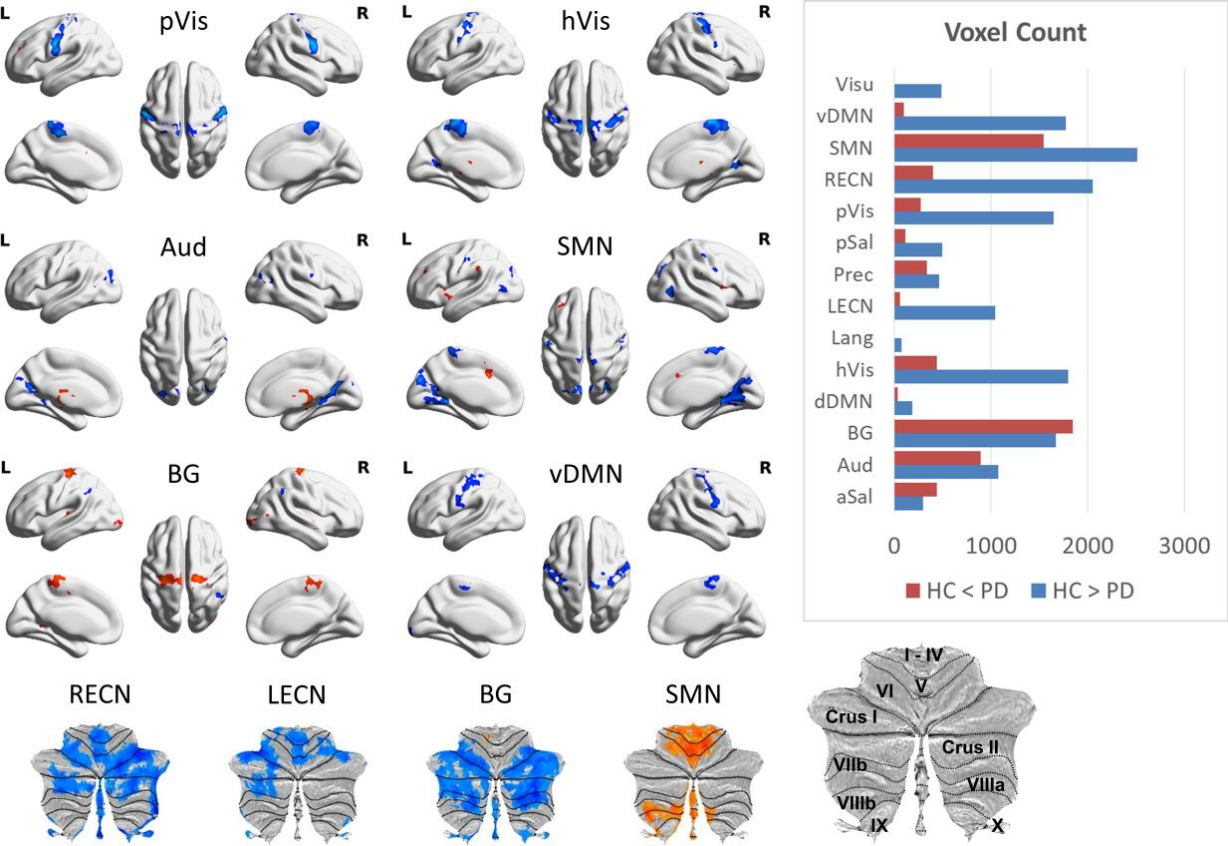
**Regions with significant connectivity alterations with several RSNs**. Highlighted regions indicate significant (FWEc *P* < 0.05; CDT *P* = 0.001) alterations in FCOR values with some representative resting-state networks between patient and control groups. Regions shown in blue have significantly lower FCOR values in patients with Parkinson's disease compared with controls, whereas those in red have significantly higher FCOR values in the patient group. The horizontal bar plot (inset) showed the number of voxels affected in each RSN.

Results of the two-sample *t*-tests of whole-brain FCOR values between control and patient groups are summarized in Fig. 1. Among the networks examined, SMN has the highest number of voxels showing significantly lower FCOR values in the patient group compared with controls, followed by RECN, then high visual network (hVis), ventral default mode network (vDMN), BG, primary visual network (pVis), Aud, LECN, and others. On the other hand, BG has the highest number of voxels showing significantly higher FCOR values in the patient group compared with controls. This is followed by SMN, Aud, hVis, and others. Most of the connectivity alterations were mainly observed in primary processing systems (SMN, pVis, hVis, and Aud), BG, (L/R) ECN, and vDMN. Connections among primary processing networks were also severely affected. Specifically, connections from sensorimotor regions to the visual networks (pVis, hVis), as well as from visual regions to SMN and Aud were significantly reduced in the patient group. In contrast, BG was characterized by higher connections to several sensorimotor regions but lower to cerebellar regions. The connectivity of several sensorimotor regions to vDMN was also lower in the patient group. Similarly, the connectivity of widespread cerebellar regions to (L/R) ECN was significantly lower in the patient group, whereas that to the SMN was higher. The list of regions showing significant difference in FCOR values between patients and controls for all RSNs is given in Supplementary Table 1. Contrast maps for the rest of the RSNs not shown in Fig. 1 are given in Supplementary Fig. 1.

### Altered connectivity of sensorimotor and cerebellar connector hubs

**Figure 2:**
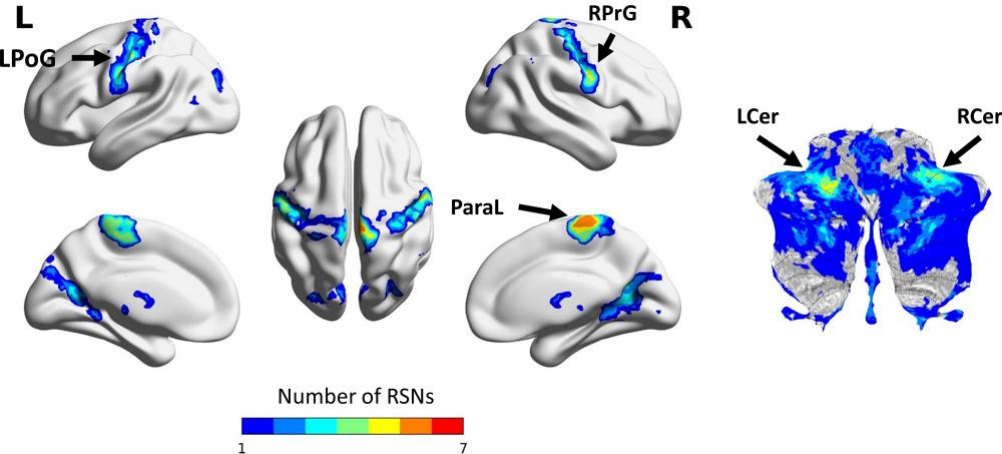
**Regions with altered FCOR values across multiple RSNs.** Summary map showing regions with significant decrease in FCOR values in several RSNs in patients compared with the control group. Voxel values indicate the number of RSNs with significant difference in FCOR values between the two groups. Black arrows indicate top five connector hubs located in the sensorimotor cortex and cerebellum with significant connectivity alterations to most RSNs.

**Figure 3:**
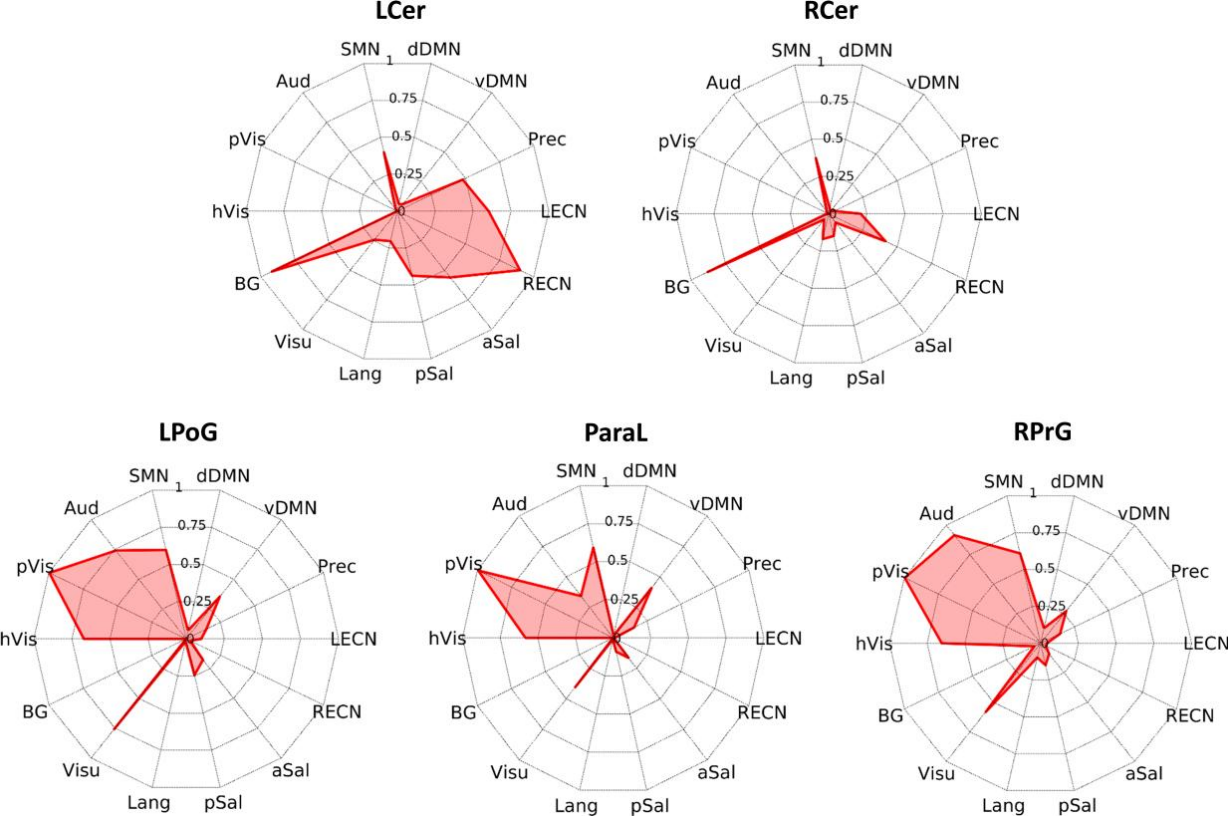
**Connectivity profile of the identified connector hubs in healthy controls.** Using each connector hub as seed ROI, individual FC maps were constructed using data from the healthy control group. For each ROI, a group-level FC map was generated using a one-sided one-sample *t*-test of the constructed individual FC maps. A threshold value of *P* < 0.05, corrected for multiple comparisons using family-wise error correction at the cluster level with a CDT set to *P* = 0.001, was applied to the resulting statistical maps. FCOR values associated with the 14 RSNs were then estimated using the group-level FC maps and are shown in the displayed spider plots.

**Figure 4:**
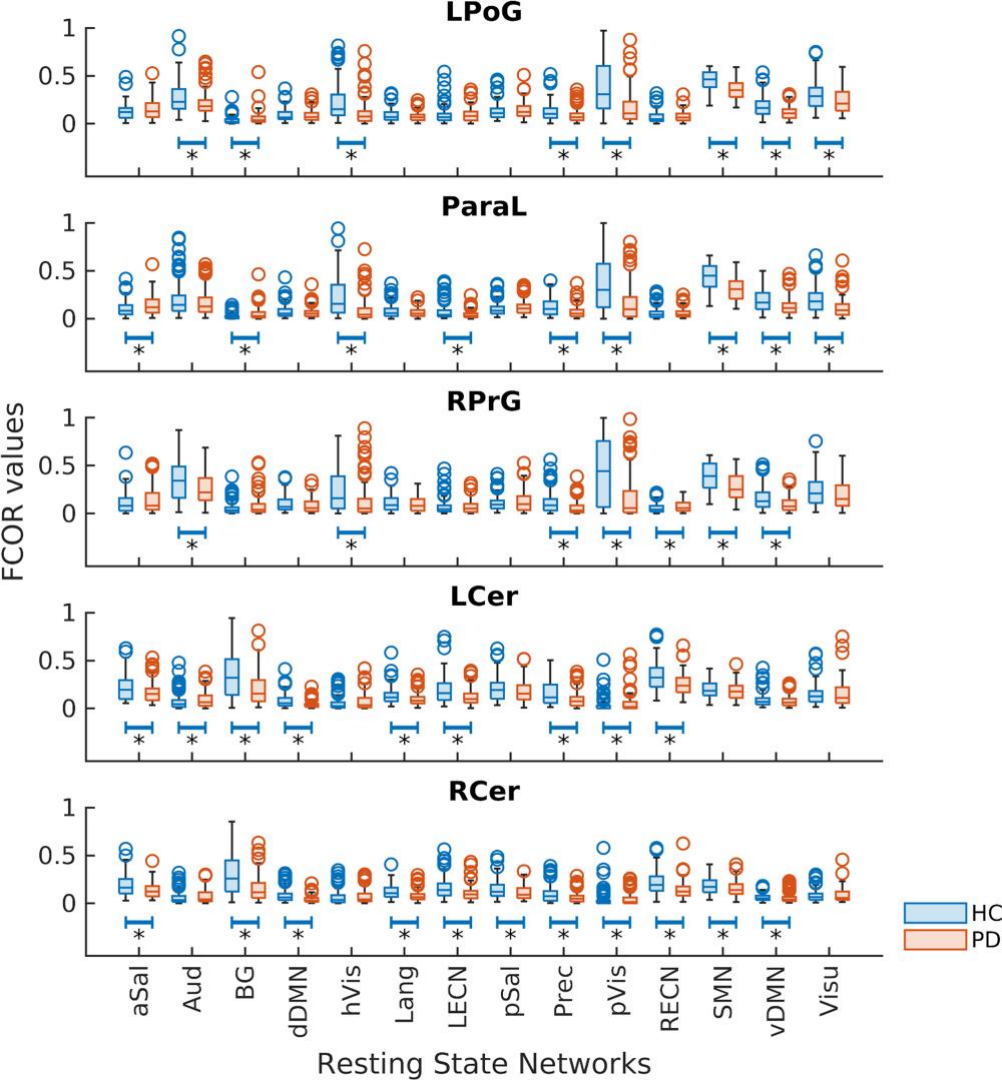
**Comparison of FCOR values of the identified connector hubs between patients and controls.** Boxplots of FCOR values associated with the 14 resting-state networks of the affected connector hubs in the sensorimotor and cerebellar regions indicated by black arrows in Fig. 2. Mean FCOR values within the identified connector hubs were extracted from the individual FCOR maps of the 14 RSNs in all participants and summarized in the displayed boxplots. For each RSN in each connector hub, differences in these mean FCOR values between patient and control groups were evaluated using a non-parametric rank sum test and the significance evaluated using FDR *q* < 0.05. RSNs exhibiting significant difference in FCOR values between patients and controls are indicated by the ‘*’ symbol. Actual FDR-corrected *P*-values are given in Supplementary Table 2

Regions that showed significantly lower FCOR values in the patient group compared with the control group across all RSNs are summarized in Fig. 2. Colour map indicates the number of RSNs where the voxel’s connectivity were significantly altered. A limited number of regions, mainly located in the sensorimotor cortex and cerebellum, have altered FCOR values to multiple RSNs. These regions, indicated by black arrows, are located in the left postcentral gyrus (LPoG), right precentral gyrus (RPrG), paracentral lobule (ParaL), posterior left cerebellum (LCer), and anterior right cerebellum (RCer). Seed-based connectivity analyses showed that these regions were significantly connected to multiple RSNs in healthy controls (Fig. 3), demonstrating that these regions are indeed connector hubs. Specifically, cerebellar connector hubs have significant connections with BG, SMN, control (ECN and Sal) and default mode (Prec) networks. On the other hand, sensorimotor connector hubs were primarily connected with primary processing networks (Vis, Aud, and SMN), as well as Visu and vDMN. Comparing FCOR values of these connector hubs between patient and control groups, sensorimotor connector hubs have significantly lower FCOR values in primary processing networks (Aud, (p/h) Vis, and SMN) but significantly higher values in BG (Fig. 4). Other networks including Prec, vDMN, Visu, aSal, and ECN, were also affected. On the other hand, alterations in the two cerebellar connector hubs (LCer, RCer) mostly involved significantly lower connectivity with the core neurocognitive networks (Sal, DMN, ECN) and BG, although higher connectivity to pVis from L/RCer connector hubs, to Aud from LCer, and to SMN from RCer were also observed.

### High predictive values of connector hubs’ FCOR features

Using FCOR values of the identified connector hubs as features, we examined its predictive value using linear SVMs. Here, FCOR values to all RSNs, not just those with significant difference, of the five connector hubs were used as features (total of 70 features per participant). The resulting 10-fold cross validation accuracies are summarized in Table 2. The ParaL’s FCOR values have the highest predictive value of 73.23% in classifying patients from controls. This is followed by the FCOR values of RPrG at 70.20%, then LPoG at 69.70%, LCer at 63.64%, and finally RCer at 62.63%. Combining the FCOR values of sensorimotor connector hubs (LPoG, RPrG, and ParaL) lead to a jump in the classification accuracy to 75.76%, whereas that of the cerebellar connector hubs (L/R Cer) to 65.15%. Overall accuracy using FCOR values from all connector hubs was 73.74%.

**Table 2:** SVM classification performance

ROI	Accuracy, % (each ROI)	Accuracy, % (regional)	Accuracy, % (All)
LCer	63.64	65.15	73.74
RCer	62.63		
LPoG	69.70	75.76	
ParaL	73.23		
RPrG	70.20		

LCer = left cerebellum; LPoG = left postcentral gyrus; ParaL = paracentral lobule; ROI = region-of-interest; RCer = right cerebellum; RPrG = right precentral gyrus; SVM = support vector machines.

### FCOR values contributing to the highest variation in connectivity in patients

**Figure 5:**
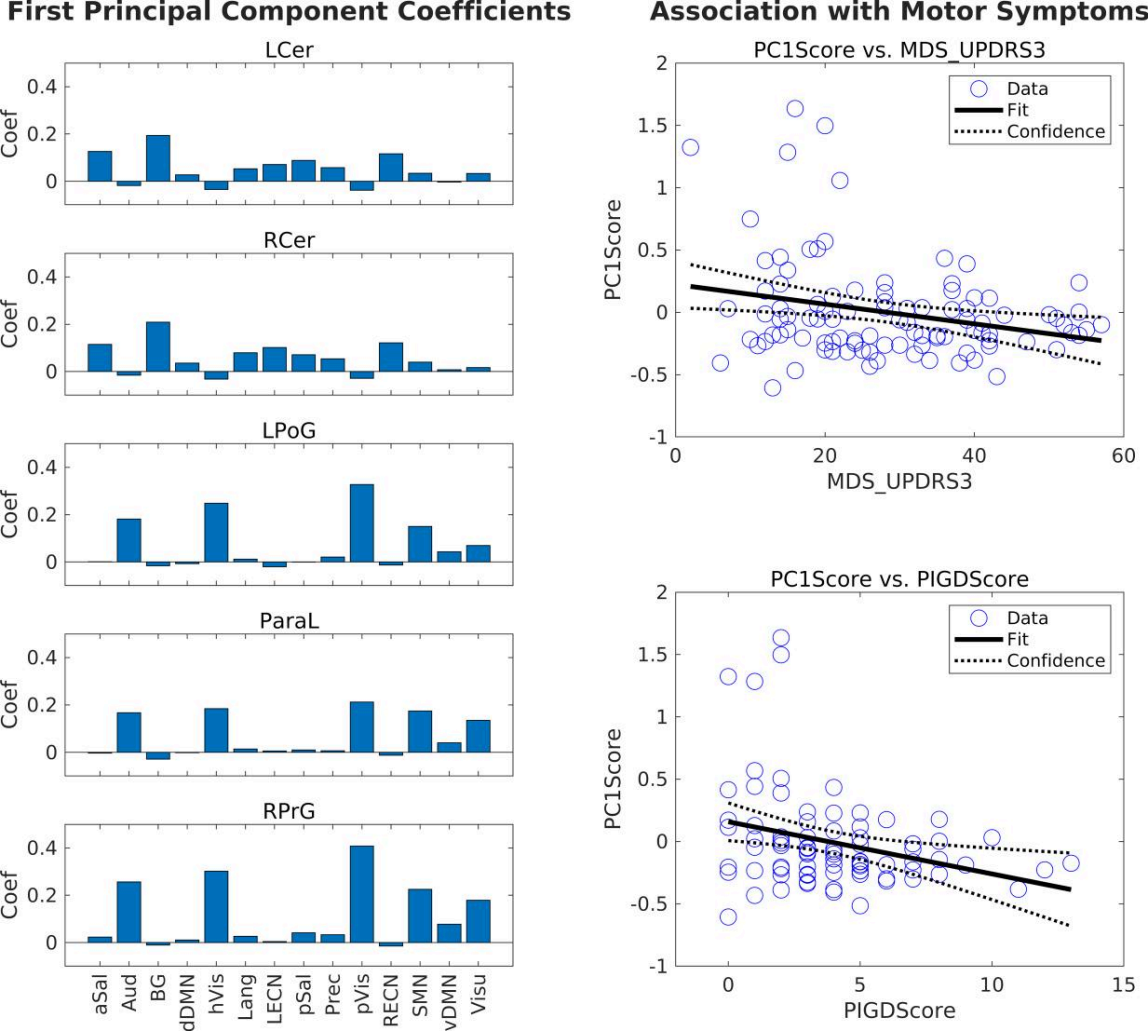
**First principal component of the identified connector hubs’ FCOR values and its association with motor symptoms.** Bar plots on the left showed the coefficients associated with the first principal component. For this analysis, mean FCOR values within the identified connector hubs were extracted from individual FCOR maps of the 14 RSNs in the patient group. Using these mean FCOR values as features, independent component analysis was performed and the first principal component, represented by its associated coefficients (shown in the left bar plots), as well as the corresponding component scores were extracted. For the cerebellar connector hubs, FCOR values associated with salience, basal ganglia, and executive control networks have higher weights compared to other networks. On the other hand, for sensorimotor connector hubs, FCOR values associated with the primary processing networks (visual, auditory, and sensorimotor), as well as ventral default mode and visuospatial networks are weighted more than the other RSNs. The first principal component's scores were also associated with patients’ motor symptoms, quantified using Pearson's correlation coefficient *r*, as shown in the right plots. The top plot showed the association between component scores and MDS-UPDRS Part III total scores (*r* = –0.2586, *P* = 0.0098), whereas the bottom plot showed the association between component scores and PIGD score (*r* = –0.2911, *P* = 0.0088)

We further performed principal component analysis to identify specific FCOR values of the identified connector hubs contributing to the highest variance in connectivity in the patient group. Weights associated with the first principal component is plotted in Fig. 5. For the cerebellar connector hubs, FCOR values associated with Sal, BG, and ECN networks were weighted higher compared with that of the other networks. On the other hand, for sensorimotor connector hubs, FCOR values associated with the primary processing networks (Vis, Aud, and SMN), as well as that of vDMN and Visu have higher weights than the other networks. The estimated component scores associated with the first principal component also correlated with MDS-UPDRS Part III total score (*r* = –0.2586, *P* = 0.0098) as well as with the PIGD score (*r* = –0.2911, *P* = 0.0088). Raw data and regression plots are shown in the right column of Fig. 5.

### Association between connector hubs’ FCOR values and motor scores

In terms of the association between FCOR values and motor scores, we found correlation between FCOR values associated with BG network and MDS-UPDRS Part III total score for both cerebellar connector hubs (LCer: *r* = –0.237, *P* = 0.0183; RCer: *r* = –0.271, *P* = 0.0066). For sensorimotor connector hubs, LPoG’s FCOR values with hVis (*r* = –0.246, *P* = 0.0140), ParaL’s FCOR values with Visu (*r* = –0.242, *P* = 0.0157), and RPrG’s FCOR values with pVis (*r* = –0.247, *P* = 0.0139) also correlated with MDS-UPDRS Part III total score.

**Figure 6:**
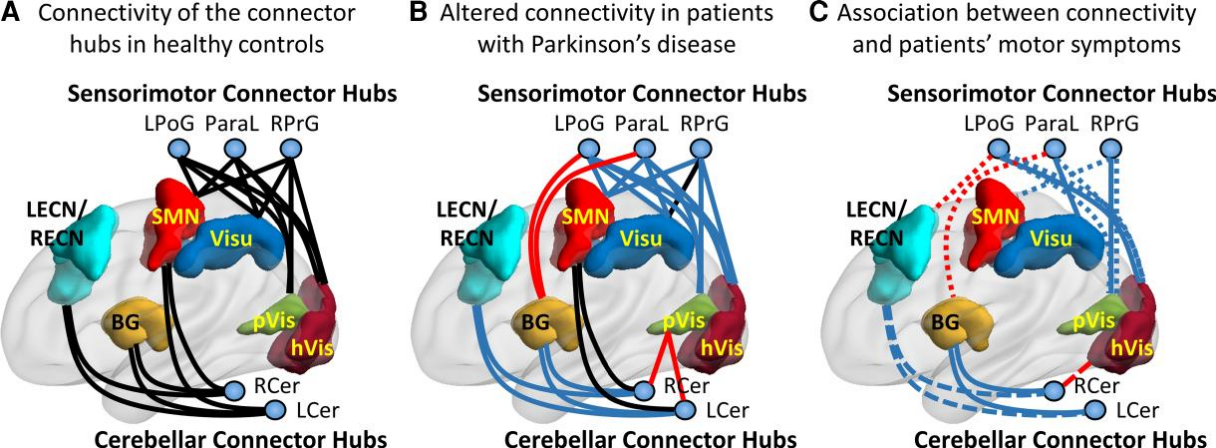
**Graphical summary of results.** (**A**) Connectivity of the different connector hubs to different resting-state networks in healthy controls. (**B**) Alterations in the connectivity of connector hubs in the patient group compared to controls. Blue, red, and black lines indicated lower, higher, and no significant difference in connectivity, respectively, in the patient group compared to the control group. (**C**) Association between connectivity values and clinical data. Solid lines represent association with MDS-UPDRS Part III total score, dashed lines with tremor score, and dotted lines with PIGD score. Blue colour indicates negative association, whereas red colour indicates positive association. Note that for simplicity not all resting-state networks, and only representative nodes for some of the shown networks, are displayed.

Tremor scores mainly correlated with cerebellar connector hubs’ FCOR values associated with executive control and default mode networks, whereas PIGD scores primarily correlated with sensorimotor connector hubs’ FCOR values associated with primary processing networks. Specifically, tremor scores correlated with LCer’s FCOR values with LECN (*r* = –0.250, *P* = 0.025), RCer’s FCOR values with dDMN (*r* = –0.288, *P* = 0.009), LECN (*r* = –0.320, *P* = 0.004), Prec (*r* = –0.223, *P* = 0.047), RECN (*r* = –0.277, *P* = 0.013), and hVis (*r* = 0.223, *P* = 0.046), as well as LPoG’s FCOR values with dDMN (*r* = –0.231, *P* = 0.039). On the other hand, PIGD scores correlated with LPoG’s FCOR values with hVis (*r* = –0.250, *P* = 0.025), pVis (*r* = –0.371, *P* = 0.001), and LECN (*r* = 0.274, *P* = 0.014), ParaL’s FCOR values with BG (*r* = 0.230, *P* = 0.040) and hVis (*r* = –0.234, *P* = 0.037), as well as RPrG’s FCOR values with pVis (*r* = –0.332, *P* = 0.003) and SMN (*r* = –0.278, *P* = 0.013). These associations are summarized in Fig. 6C.

Finally, we also observed correlation of FCOR values with LEDD mostly in the sensorimotor connector hubs. Specifically, LEDD correlated with LPoG’s FCOR values with Visu (*r* = 0.215, *P* = 0.032), ParaL’s FCOR values with Lang (*r* = –0.273, *P* = 0.006), Prec (*r* = 0.364, *P* = 2.088×10^–4^), pVis (*r* = 0.278, *P* = 0.005), and vDMN (*r* = 0.267, *P* = 0.008), RPrG’s FCOR values with BG (*r* = –0.203, *P* = 0.044) and pVis (*r* = –0.203, *P* = 0.043), as well as LCer’s FCOR values with dDMN (*r* = 0.204, *P* = 0.043). Note that the indicated *P*-values were uncorrected.

## Discussion

In this study, we used a hypothesis-free approach to identify connector hub regions involved in Parkinson’s disease and the associated connectivity alterations of these hubs with several large-scale brain networks. For this, we extensively examined connectivity alterations for every voxel in the brain with several large-scale functional networks using a network metric we recently developed called FCOR. First, analysis at the level of 14 different RSNs showed that in Parkinson’s disease, several networks including primary processing (pVis, hVis, SMN, Aud), control (RECN, LECN, Visu), BG, and default mode (vDMN) have significant connectivity alterations in the visual and sensorimotor areas as well as in the cerebellum. Of particular interest is the BG and SMN, which showed reduced or enhanced connectivity with these regions. In BG, connections with the posterior cerebellar lobe were mainly attenuated, whereas connections with the sensorimotor cortex were enhanced. On the other hand, SMN showed weakened connections within the sensorimotor and visual areas and enhanced connections with the basal ganglia and anterior cerebellar lobes. Next, among affected regions across several RSNs, we have identified a small number of connector hubs mainly localized in the sensorimotor cortex and the cerebellum (Fig. 6). Sensorimotor connector hubs (LPoG, ParaL, and RPrG), which connect with primary processing networks (Aud, SMN, and Vis), Visu, and vDMN in healthy controls, and cerebellar connector hubs (LCer and RCer), which connect with the BG, SMN, control (Sal, ECN), and default mode (Prec) networks (Fig. 6A), exhibited significant connectivity impairment across multiple resting-state functional networks in Parkinson’s disease (Fig. 6B). In addition, the impaired connectivity of these connector hubs was associated with the severity of motor dysfunction (Fig. 6C). Specifically, connectivity of the cerebellar connector hubs to the BG was associated with MDS-UPDRS Part III total score and to the control (LECN, RECN) and default mode (dDMN, Prec) networks with the tremor dominant score. In contrast, connectivity of the sensorimotor connector hubs to the Visu and visual networks was associated with MDS-UPDRS Part III total score and to the primary processing networks (Vis, SMN) and BG with the PIGD score. These connector hubs have also FCOR values that were highly predictive in classifying patients from healthy controls with an accuracy of 75.76% for the sensorimotor connector hubs and 65.15% for the cerebellar connector hubs. Taken together, these findings suggest that, together with the basal ganglia, cerebellar and sensorimotor connector hubs are significantly involved in Parkinson’s disease with their altered connectivity potentially driving the varying clinical manifestations typically observed in this disorder.

Parkinson’s disease has been thought to be caused by damage to the extrapyramidal system, resulting in motor slowness, muscle rigidity, and tremor. Levodopa therapy ameliorates Parkinsonism but does not always lead to complete recovery. Although basal ganglia dysfunction is invariably at the heart of Parkinson’s disease, our results showed that the sensorimotor cortex and cerebellum are also extensively involved. This is consistent with recent evidence supporting the view that bradykinesia in Parkinson’s disease may involve the sensorimotor cortex and cerebellum.^54^ Cerebellar involvement has also been reported in dopamine-resistant tremor patients.^55^ Mounting evidence have also shown the involvement of sensorimotor cortex, basal ganglia, and cerebellum in motor functions such as motor learning, model-based control, and model-free exploration. As suggested by Doya,^56^ the basal ganglia, central to reinforcement learning, and the cerebellum, central to supervised learning, could support unsupervised learning in the cerebral cortex. This learning-oriented functional interpretation of these systems could provide a framework in understanding the complementary roles these systems play in motor control. In addition, although the cerebellum and basal ganglia were previously considered to be distinct subcortical systems, accumulating evidence have shown that the cerebellum, the basal ganglia, and the cerebral cortex formed an integrated network.^57,58^ This perspective could help explain how abnormal activity in one system could lead to network-wide effects.

Based on the connector hubs’ association with clinical data, our findings suggest that dysfunction of the sensorimotor connector hubs, particularly supplementary motor area (SMA), may be associated with PIGD subtype. The SMA, where one of the sensorimotor connector hubs is located, is critical in initiating movements, particularly those that are internally generated.^59,60^ Increasing evidence also suggests that SMA plays a critical role in the pathogenesis of freezing of gate (FOG),^61–63^ one of the most common debilitating features of Parkinson’s disease. Using transcranial magnetic stimulation (TMS) over SMA showed significant improvement in FOG symptoms, whereas no improvement where found in the sham group.^64,65^ The observed improvement was also attributed to the normalization of the abnormal connectivity pattern associated with FOG, as well as the overall connectivity pattern disruption in Parkinson’s disease.^65^ In a randomized trial targeting both primary motor (M1) and dorsolateral prefrontal cortex (dlPFC) for TMS treatment, improvement in motor symptoms was only observed when M1 was the target, but no additional benefits were observed when both M1 and dlPFC were used as targets.^66^ Thus, the impairment of the sensorimotor connector hubs may be likely to be an important factor underlying PIGD phenotype in Parkinson’s disease.

While the sensorimotor connector hubs may be associated with akinesia/rigidity subtype, the tremor dominant subtype may be more associated with the dysfunction of cerebellar connector hubs. There is a growing evidence that cerebello-thalamic-cortico (CTC) circuit plays an important role in the pathophysiology of the parkinsonian resting tremor.^67–69^ For instance, an optimal target for tremor treatment using deep brain stimulation is the thalamic ventral intermediate nucleus, which is the cerebellar territory of the thalamus. In a study investigating dopamine-resistant and dopamine-responsive tremor in patients with Parkinson’s disease,^55^ both groups showed activity in the CTC circuit; however, the resistant group showed increased tremor-related activity in the cerebellum, whereas the responsive group in the thalamus and secondary somatosensory cortex. Levodopa inhibited thalamic activity in both groups but was more significant in the responsive group, suggesting more cerebellar influences in the cerebellar thalamus in the dopamine-resistant group. Our results also showed that FCOR values of cerebellar connector hubs to the BG are associated with MDS-UPDRS Part III total score and that to the executive control and default mode networks with the tremor score, suggesting the contribution of these hubs to this motor dysfunction.

In addition to motor symptoms, we also observed disconnection among primary processing networks in the patient group. In healthy participants, primary processing networks are closely linked with each other.^37^ For instance, some regions in the sensorimotor network have shown strong FCOR values with regions in the visual network and vice versa. This strong interconnection among these networks is prominently missing in the patient group. Intriguingly, visual cueing methods are commonly applied to improve gait freezing in patients with Parkinson’s disease.^70^ Art therapy has been shown to improve visual-cognitive skills and visual exploration strategies as well as general motor functions in Parkinson’s disease.^71^ Moreover, these improvements were associated with increased connectivity within primary and associative visual networks. Our results indicating impaired connections of sensorimotor connector hubs to primary processing networks including visual and sensorimotor suggest that the integration of various information from these networks may be significantly affected in patients with Parkinson’s disease.

In a previous study, we have also reported that reduced FC of the posterior cerebellar lobes is associated with non-memory-type cognitive decline^13^ and that reduced FC between the cerebellum and basal ganglia is associated with parkinsonism and cognitive decline.^40^ Several recent studies have also implicated cerebellum involvement in Parkinson’s disease. For instance, attention deficits associated with cerebellar lesions are associated with visual hallucinations.^72^ Action observation training and motor imagery also improved motor learning in Parkinson’s disease with increased cerebellar activity observed using functional MRI.^73^ In this regard, further investigation of the role of the cerebellum, particularly the identified cerebellar connector hubs, in the pathogenesis of Parkinson’s disease from a network perspective may lead to new biomarkers and therapeutic targets for the cerebellum, as well as the elucidation of the pathogenesis of drug-resistant tremor.

In terms of potential biomarkers, our result showing high predictive power of the FCOR values of these connector hubs could provide a more specific and sensitive biomarker for diagnostic, prognostic, treatment, and disease-monitoring purposes. The accessibility of the sensorimotor connector hubs make them good candidates as targets for non-invasive treatment of Parkinson’s disease such as TMS or electroconvulsive therapy. Although there has been differences in the efficacy of TMS to treat motor symptoms in Parkinson’s disease, this may be due to the treatment target site. Our findings suggest that the identified connector hubs are promising potential targets for these therapies. We do note that the reliability and accuracy of this metric still remains to be tested in independent samples of patients.

Finally, the present study was conducted without stopping anti-Parkinsonian drugs. This was necessary to analyze the pathogenesis of Parkinson’s disease in real world situation and to consider the effect of long-duration response to levodopa.^74^ However, we do note that dopaminergic therapy in Parkinson’s disease had been shown to normalize observed increased connectivity in unmedicated patients.^75^ Decrease in network connectivity within SMN and between SMN and other networks was also more pronounced after discontinuation of levodopa with connectivity decreases partially normalizing during ON state.^26^ Correlation between LEDD and FCOR values was observed in this study. Thus, this study’s findings should be interpreted under ON state condition.

In summary, using a novel network metric called FCOR to extensively assess connectivity alterations in patients with Parkinson’s disease, we have identified connector hubs located mainly in the cerebellum and sensorimotor regions with connections to multiple large-scale functional networks that were significantly impaired. FCOR values of these connector hubs correlated with clinical scores and provided higher predictive values in classifying patients from healthy controls.

## Supplementary Material

fcac214_Supplementary_Data

## Abbreviations

ACE-R =: Addenbrooke’s Cognitive Examination—Revised
aSal =: anterior salience network
Aud =: auditory network
BG =: basal ganglia network
CDT =: cluster defining threshold
CSF =: cerebrospinal fluid
CTC =: cerebello-thalamic-cortico circuit
dDMN =: dorsal default mode network
dlPFC =: dorsolateral prefrontal cortex
FC =: functional connectivity
FCOR =: functional connectivity overlap ratio
FDR =: false discovery rate
FOG =: freezing of gait
FOV =: field of view
FWEc =: family-wise error correction at the cluster level
HCs =: healthy controls
hVis =: high visual network
Lang =: language network
LCer =: left cerebellum
LECN =: left executive control network
LEDD =: levodopa equivalent daily dose
LPoG =: left postcentral gyrus
M1 =: primary motor cortex
MDS-UPDRS =: Movement Disorder Society Unified Parkinson’s Disease Rating Scale
MNI =: Montreal Neurological Institute
ParaL =: paracentral lobule
PIGD =: postural instability and gait difficulty
Prec =: precuneus network
pVis =: primary visual network
RCer =: right cerebellum
RECN =: right executive control network
ROI =: region-of-interest
RPrG =: right precentral gyrus
rsfMRI =: resting-state functional MRI
RSN =: resting-state network
SD =: standard deviation
SMA =: supplementary motor area
SMN =: sensorimotor network
SVM =: support vector machine
TE =: echo time
TI =: inversion time
TMS =: transcranial magnetic stimulation
TR =: repetition time
vDMN =: ventral default mode network
Visu =: visuospatial network
WM =: white matter
