# Supplementary material for: Connectivity impairment of cerebellar and sensorimotor connector hubs in Parkinson’s disease
Source: Brain Commun. 2022 Aug 20;4(5):fcac214. doi: 10.1093/braincomms/fcac214 (PMC9438962; doi:10.1093/braincomms/fcac214)
Supplement: fcac214_Supplementary_Data [file fcac214_supplementary_data.pdf]

Connectivity impairment of cerebellar and sensorimotor connector hubs in Parkinson's disease

Epifanio Bagarinao, Kazuya Kawabata, Hirohisa Watanabe, Kazuhiro Hara, Reiko Ohdake, Aya Ogura, Michihito Masuda, Toshiyasu Kato, Satoshi Maesawa, Masahisa Katsuno, Gen Sobue

Supplementary Materials

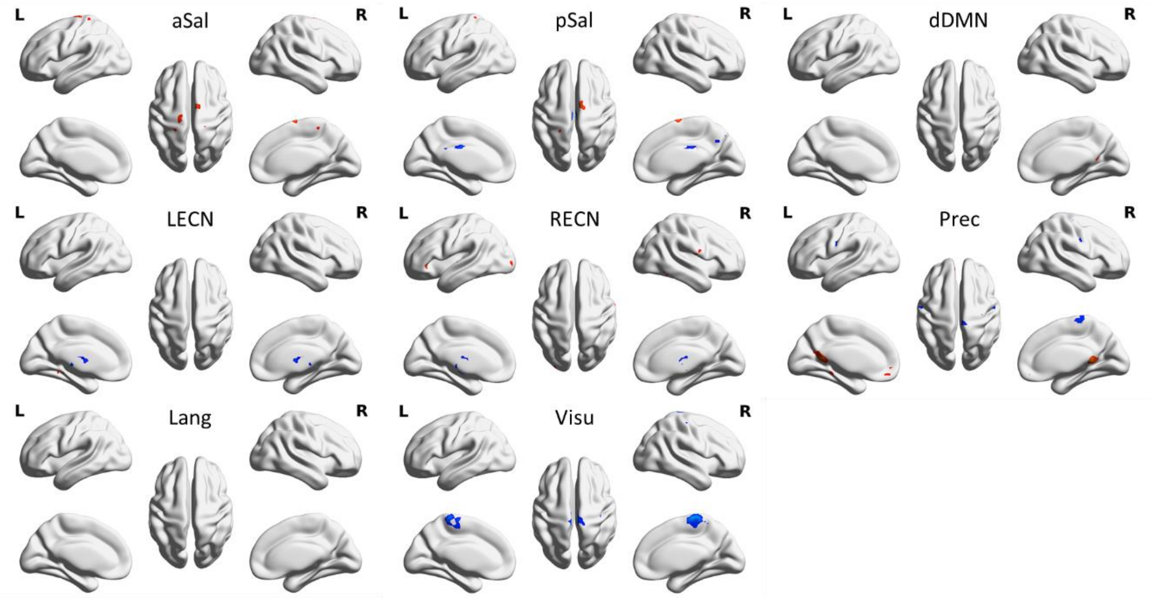

Supplementary Figure 1. Regions with significant connectivity alterations with several RSNs. Highlighted regions indicate significant (FWEc $p < 0.05$; CDT $p = 0.001$) alterations in FCOR values between patient and control groups. Regions shown in blue have significantly lower FCOR values in patients with Parkinson's disease compared to controls, whereas those in red have significantly higher FCOR values in the patient group.

Supplementary Table 1. Regions showing significant difference in FCOR values between patients and controls

Contrast	X	Y	Z	z-Value	Cluster Size	Area	Other Peaks
**Anterior Salience Network**							
HC > PD	3	-24	-21	4.88	48	Brainstem	
	27	-54	-33	4.45	67	R Cer	
	15	-72	-24	4.4	133	R Cer	
	0	60	42	4.15	48	L MSFG	
HC < PD	-15	-12	78	5.46	192	L PrG	L PoG
	9	-3	69	5.04	33	R SFG	
	-39	-27	-9	4.81	35	L Hip	
	15	-51	75	4.61	64	R SPL	R MPoG, R PoG
	27	-27	75	4.55	71	R PoG	R PrG
	36	-33	0	4.06	42	R Hip	
**Auditory Network**							
HC > PD	24	-66	21	6.43	1001	R Cun	L Cun, R PCu
	-15	-45	0	4.66	41	L LiG	L PCgG
	57	-6	27	3.88	32	R PoG	
HC < PD	-21	24	-3	5.4	713	L Cau	
	36	-27	-3	4.61	75	R Hip	
	-39	-36	-3	4.6	104	L Hip	
**Basal Ganglia Network**							
HC > PD	24	-72	-45	6	1198	R Cer	L Cer
	51	-60	51	4.93	228	R AnG	
	-51	-51	54	4.66	211	L SMG	L AnG
	18	-36	18	4.31	30	R ThP	R MCgG, R PCgG
HC < PD	-27	-24	63	5.44	1091	L PrG	R PrG
	51	0	-9	5.11	149	R PP	R CO, R TTG
	24	-48	3	4.69	52	R LiG	R Calc
	-51	-3	-9	4.52	138	L STG	L TTG, L PT
	36	-90	3	4.47	155	R IOG	R OCP
	-21	-48	3	4.39	89	L LiG	
	69	-6	12	4.05	28	R PoG	R PrG
	-30	-96	-18	3.94	88	L IOG	
	-18	-75	0	3.94	51	L Calc	L LiG

**Dorsal Default Mode Network**							
HC > PD	-24	-63	-27	4.91	93	L Cer	
	27	-54	-33	4.05	63	R Cer	
	63	-48	45	3.76	31	R SMG	
HC < PD	9	-54	12	4.32	35	R PCu	
**High Visual Network**							
HC > PD	6	-24	72	6.24	1129	R MPrG	L PrG
	51	-15	51	5.54	359	R PoG	R PrG
	18	-57	9	4.88	158	R PCu	R Cun
	-18	-75	12	4.39	120	L Calc	L PCu, L Cun
	54	-3	30	3.95	31	R PrG	R PoG
HC < PD	12	-6	15	4.89	245	R ThP	L ThP
	18	-93	-36	4.33	32	R Cer	
	-6	-18	-6	4.32	47	L ThP	R ThP
	-6	-84	-30	4.31	62	L Cer	R Cer
	42	24	9	4.1	54	R FO	R OpIFG, R TrIFG
**Language Network**							
HC > PD	36	-54	-30	4.06	36	R Cer	
	-30	-51	-30	3.73	38	L Cer	
**Left Executive Control Network**							
HC > PD	-15	-9	18	5.63	494	L ThP	L Cau
	-30	-51	-30	5.13	296	L Cer	
	-3	-48	-12	4.59	131	L Cer	R Cer
	42	-54	-30	4.35	123	R Cer	
HC < PD	-27	-36	-9	4.28	62	L Hip	L FuG, L LiG
**Precuneus Network**							
HC > PD	-18	15	9	4.64	111	L Cau	L Pu
	21	3	0	4.59	77	R Pal	R Cau
	9	-27	81	4.48	80	R PrG	R MPrG
	51	-6	30	4.34	48	R PrG	R PoG
	39	-27	66	4.22	30	R PoG	R PrG
	-24	-54	-30	4.08	42	L Cer	
	-57	-9	21	4	44	L PoG	
	-51	-24	57	3.75	29	L PoG	L PrG
HC < PD	6	-45	6	5.39	266	R PCgG	R PCu, L PCu
	-21	-39	-12	4.64	36	L PHG	
	-6	51	-3	3.77	32	L MSFG	L MFC, R MFC

**Posterior Salience Network**							
HC > PD	-3	-15	30	4.96	81	L MCgG	R MCgG
	0	54	45	4.56	69	L MSFG	R SFG
	33	-84	-36	4.24	143	R Cer	
	6	-60	36	4.05	46	R PCu	
	12	-87	-27	3.99	29	R Cer	
	-30	-87	-39	3.98	53	L Cer	
	15	-51	-33	3.88	40	R Cer	
	15	9	6	3.87	34	R Cau	R Pal
HC < PD	12	-6	72	5.22	47	R SFG	
	21	3	54	4.44	36	R SFG	
	-24	-39	75	4.27	28	L PoG	
**Primary Visual Network**							
HC > PD	39	-15	39	6.6	1649	R PoG	L PoG
HC < PD	24	-42	15	4.93	36	R Hip	
	-15	30	9	4.36	46	L Cau	L ACgG
	-27	45	24	4.29	43	L MFG	
	15	30	9	4.28	33	R Cau	
	6	-51	-6	4.21	51	R LiG	
	12	3	30	4.08	38	R MCgG	L MCgG
	-9	-21	30	3.66	30	L MCgG	L PCgG
**Right Executive Control Network**							
HC > PD	6	-51	-33	6.05	987	R Cer	
	15	6	18	4.99	725	R Cau	L ThP
	-24	-54	-30	4.92	341	L Cer	
HC < PD	-45	-18	36	5.1	48	L PoG	
	-42	33	-3	4.92	67	L TrIFG	L OrIFG, L OpIFG
	51	-54	-18	4.67	70	R ITG	
	60	-6	6	4.52	68	R CO	R PP
	-57	0	0	4.51	69	L PP	L CO, L STG
	-24	-90	-12	4.34	54	L OFuG	L IOG, L LiG
	57	18	-3	3.8	27	R OpIFG	R TMP
**Sensorimotor Network**							
HC > PD	-15	-45	-3	6.31	1936	L LiG	L SOG, R SOG
	-3	-36	66	5.48	216	L MPoG	R MPrG, R PoG
	-51	-18	45	5.13	123	L PoG	
	-45	-72	6	4.97	77	L IOG	

	60	-3	33	4.43	87	R PoG	R PrG
	51	-24	54	4.36	37	R PoG	
	30	-54	57	4.01	33	R SPL	
HC < PD	12	-54	-15	5.66	439	R Cer	L Cer
	36	15	12	5.44	303	R FO	R OpIFG
	-6	15	33	5.31	120	R MCgG	R MCgG, L SMC
	-30	18	15	5.19	297	L FO	L Pu
	36	-42	9	4.78	30	R Hip	R PCu
	-24	-45	-48	4.64	147	L Cer	
	-57	-36	33	4.59	28	L SMG	
	3	-36	-54	4.45	78	Brainstem	L Cer
	54	-21	27	4.34	26	R PO	
	-36	45	27	4.21	48	L MFG	
	-6	-24	24	3.97	27	L MCgG	R MCgG
**Ventral Default Mode Network**							
HC > PD	54	-9	42	5.42	879	R PoG	R PrG
	-51	-18	48	4.91	765	L PoG	L PrG
	18	-84	6	4.32	72	R Calc	L LiG
	-9	-102	-3	4.12	56	L OCP	
HC < PD	30	-30	6	4.77	38	R ThP	R Hip
	18	30	9	4.36	36	R Cau	
	18	-36	-18	4.25	28	R Cer	R PHG
**Visuospatial Network**							
HC > PD	3	-21	69	5.66	395	R MPrG	L MPrG
	-33	-21	51	4.57	63	L PrG	L PoG
	36	-24	54	3.91	30	R PoG	R PrG

Abbreviations: AnG, angular gyrus; Calc, calcarine; Cau, caudate; Cer, cerebellum; CO, central operculum; Cun, cuneus; FCOR, functional connectivity overlap ratio; FO, frontal operculum; FuG, fusiform gyrus; HC, healthy controls; Hip, hippocampus; IOG, inferior occipital gyrus; ITG, inferior temporal gyrus; L, left; LiG, lingual gyrus; MCgG, midcingulate gyrus; MFG, middle frontal gyrus; MPoG, medial postcentral gyrus; MPrG, medial precentral gyrus; MSFG, medial superior frontal gyrus; OCP, occipital pole; OFuG, occipital fusiform gyrus; OpIFG, opercular inferior frontal gyrus; Pal, pallidum; PCgG, posterior cingulate gyrus; PCu, precuneus; PD, patients with Parkinson's disease; PHG, parahippocampal gyrus; PO, posterior operculum; PoG, postcentral gyrus; PP, planum polare; PrG, precentral gyrus; PT, planum temporale; Pu, putamen; R, right; SFG, superior frontal gyrus; SMC, supplementary motor cortex; SMG, supramarginal gyrus; SPL, superior parietal lobule; STG, superior temporal gyrus; ThP, thalamus proper; TrIFG, triangular inferior frontal gyrus; TTG, transverse temporal gyrus

Supplementary Table 2. FDR-corrected p-values of the non-parametric rank sum tests between patients and controls of the FCOR values associated with the different RSNs for the identified connector hubs shown in Figure 4.

RSNs	LPoG	ParaL	RPrG	LCer	RCer
aSal	-	0.033	-	0.013	0.003
Aud	0.0047	-	0.0053	0.021	-
BG	0.00055	7.9×10^{-6}	-	5.5×10^{-5}	1.1×10^{-5}
dDMN	-	-	-	5.5×10^{-5}	1.1×10^{-5}
hVis	8.3×10^{-8}	1.8×10^{-8}	9.5×10^{-5}	-	-
Lang	-	-	-	0.00059	2.4×10^{-5}
LECN	-	0.0028	-	0.00016	0.00015
pSal	-	-	-	-	0.013
Prec	0.0025	2.8×10^{-5}	9.5×10^{-5}	0.00081	0.01
pVis	4.8×10^{-9}	7.6×10^{-8}	6.5×10^{-8}	0.00081	0.013
RECN	-	-	0.039	0.00011	2.4×10^{-5}
SMN	8.3×10^{-8}	6.1×10^{-9}	6.7×10^{-6}	-	0.03
vDMN	1.3×10^{-5}	5.2×10^{-5}	9.5×10^{-5}	-	0.024
Visu	0.0025	2.8×10^{-7}	-	-	-

Abbreviations: -, not significant; aSal, anterior salience network; Aud, auditory network; BG, basal ganglia network; dDMN, dorsal default mode network; FDR, false discovery rate; hVis, high visual network; Lang, language network; LCer, left cerebellum; LECN, left executive control network; LPoG, left postcentral gyrus; ParaL, paracentral lobule, Prec, precuneus network; pSal, posterior salience network; pVis, primary visual network; RCer, right cerebellum; RECN, right executive control network; RPrG, right precentral gyrus; RSN, resting state networks; SMN, sensorimotor network; vDMN, ventral default mode network; Visu, visuospatial network.
